# From field to tables - a dataset of 2,449 species cultivated for food

**DOI:** 10.1016/j.dib.2026.113358

**Published:** 2026-10-17

**Authors:** Martin Davy, SAGHAS consortium, Adeline Barnaud, Cécile Berthouly-Salazar, Delphine Renard, Anne-Céline Thuillet

**Affiliations:** a DIADE, Univ Montpellier, IRD, CIRAD, Montpellier, France.; b CEFE, CNRS, EPHE, IRD, Univ Montpellier, Montpellier, France.; a DIADE, Univ Montpellier, IRD, CIRAD, Montpellier, France; b CEFE, CNRS, EPHE, IRD, Univ Montpellier, Montpellier, France

**Keywords:** Agrobiodiversity, Human uses, Traits, Food production

## Abstract

Across the vast diversity of vascular plants on Earth, around 6,000 species have been used as food sources for humans. Yet, only nine crops provide more than 60% of the calories consumed worldwide. In the face of accelerating climate change, biodiversity loss, and malnutrition, there is an urgent need to broaden the range of food crops supporting sustainable agriculture and resilient food systems.

Crop diversity plays a central role in addressing these challenges. However, effectively mobilizing this diversity requires a comprehensive understanding of its taxonomic, functional and geographical dimensions, and of its relationships with human societies through their uses and knowledge systems.

To date, data on crop species, their functional traits, human uses, and distribution remain fragmented across multiple and heterogeneous databases that vary in scope, structure, and standards. To address this gap, we developed a harmonized global dataset comprising information on 2,449 crops species. This database, called SAGHAS for “Sowing AGrobiodiversity, HArvesting Sustainability”, provides a foundation for comprehensive analyses of cultivated plant diversity (taxonomic and functional) and its biogeography.

Specifications Table

**Table:** 

Subject	Earth & Environmental Sciences
Specific subject area	Crops – plant species cultivated for food. In addition to the taxonomic dimension of these plants, information is given on their functional traits, production, human uses and geographical distributions
Type of data	Table
Data collection	All primary data were collected from different open access sources listed in Table 2. Harmonization and filters were applied to the data.
Data source location	Institut de Recherche Pour le Développement - IRD Montpellier France This dataset aggregates and harmonizes 17 different sources, described in Table 2 and in the “species_sources.tsv” table included in the dataset.
Data accessibility	Repository name: DataSuds Data identification number: https://doi.org/10.23708/LK5VUI Direct URL to data https://doi.org/10.23708/LK5VUI
Related research article	None

## 1 Value of the Data

• **Dataset of plants cultivated for food:** This dataset provides a list of 2,449 crop species with standardized taxonomic names. In addition, it compiles existing information on species’ functional traits, human uses (including and in addition to food), production as well as geographic distribution (native and introduced areas, occurrences) at sub-national levels.
• **Supporting agrobiodiversity research:** The dataset can support the identification of hotspots and coldspots of cultivated plant diversity, an often overlooked component of biodiversity as a whole. Data analyses can further help evaluate implications for adaptation to environmental changes and guide conservation strategies and breeding programs.
• **Cross-disciplinary research:** Researchers working in the domains of agriculture, ecology, ethnobotany can use this dataset to explore the geographic distribution and variation of links between crop diversity at both taxonomic and functional levels and the diversity of human uses.
• **Missing data:** Data associated with the list of 2,449 crop species are missing. Less than 5% of the species do not have information on human uses (Fig. 1). Native and introduced areas are missing for respectively 1% and 36% of the species. Occurrence information is missing for 15% of the species. 58% of the species do not have imputed traits values and 90% of species do not have production values, yield or cultivated area.

**Fig. 1:**
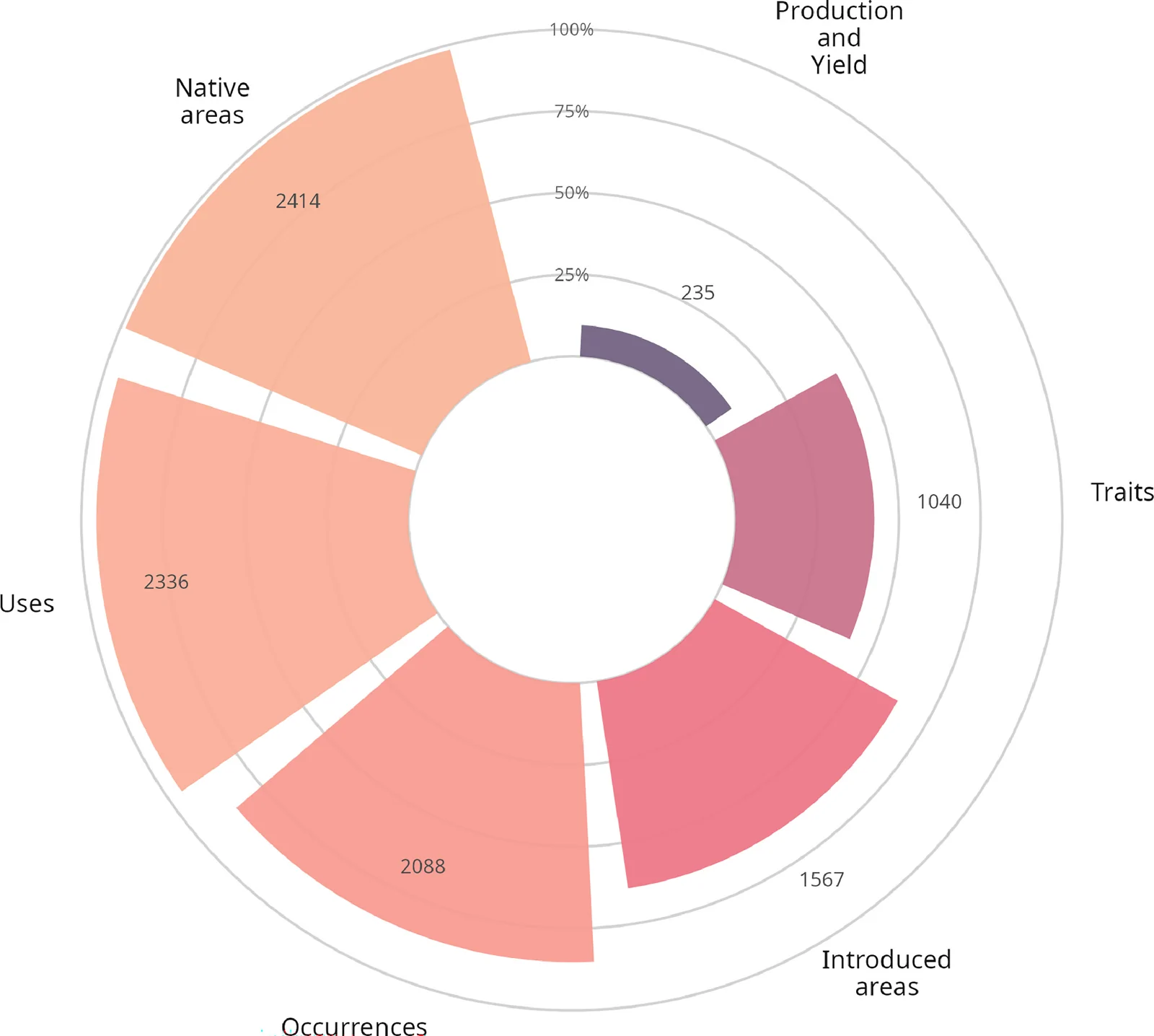
Number of species represented per data type in the SAGHAS dataset. 100% correspond to the 2,449 species selected for the SAGHAS dataset.
• **Facilitation reproducibility and data integration:** The standardized format of this dataset makes it easily interoperable with other biodiversity or agricultural datasets, encouraging its use in agroecology.

## 2 Background

Agriculture faces multiple challenges: Increasing food production, reducing environmental impacts and supporting human wellbeing. Complexity lies in balancing agronomic, social and environmental objectives under accelerating global change. Promoting crop diversity is a promising lever to achieve multiple goals. Yet, there is no global list of food crops providing harmonized data on their functional, geographical and use characteristics to help guide diversification efforts.

Existing lists vary in scope. For example Pironon et al. [1] report 35,687 used species including wild and non-food species. Conversely, the Food and Agriculture Organization publishes statistics on 235 food crops grouped into 142 categories. Additionally, lists often use incompatible taxonomic standards. The SAGHAS dataset contributes to fill this gap by providing the most comprehensive list, to date, of cultivated plants for food.

Disciplinary data on agriculture are often produced and analyzed in isolation. This significantly constrains data interoperability needed to capture the multiple dimensions of crop diversity. The present standardized list of crops allowed the compilation of data on functional traits of crops, human uses (as food and other uses), and production data. We also provide geographical data on species distribution that can further help identify centers of crop diversity and enable comparisons across scales.

## 3 Data Description

The SAGHAS dataset is composed of 10 tables (Fig. 2). Each table is described hereafter. All these tables are interconnected through the identifiers *saghas_id* and *source_id*. Each species is associated with a unique *saghas_id* (table “species”) while sources are linked via a unique *source_id* (table “species_sources”).

**Fig. 2:**
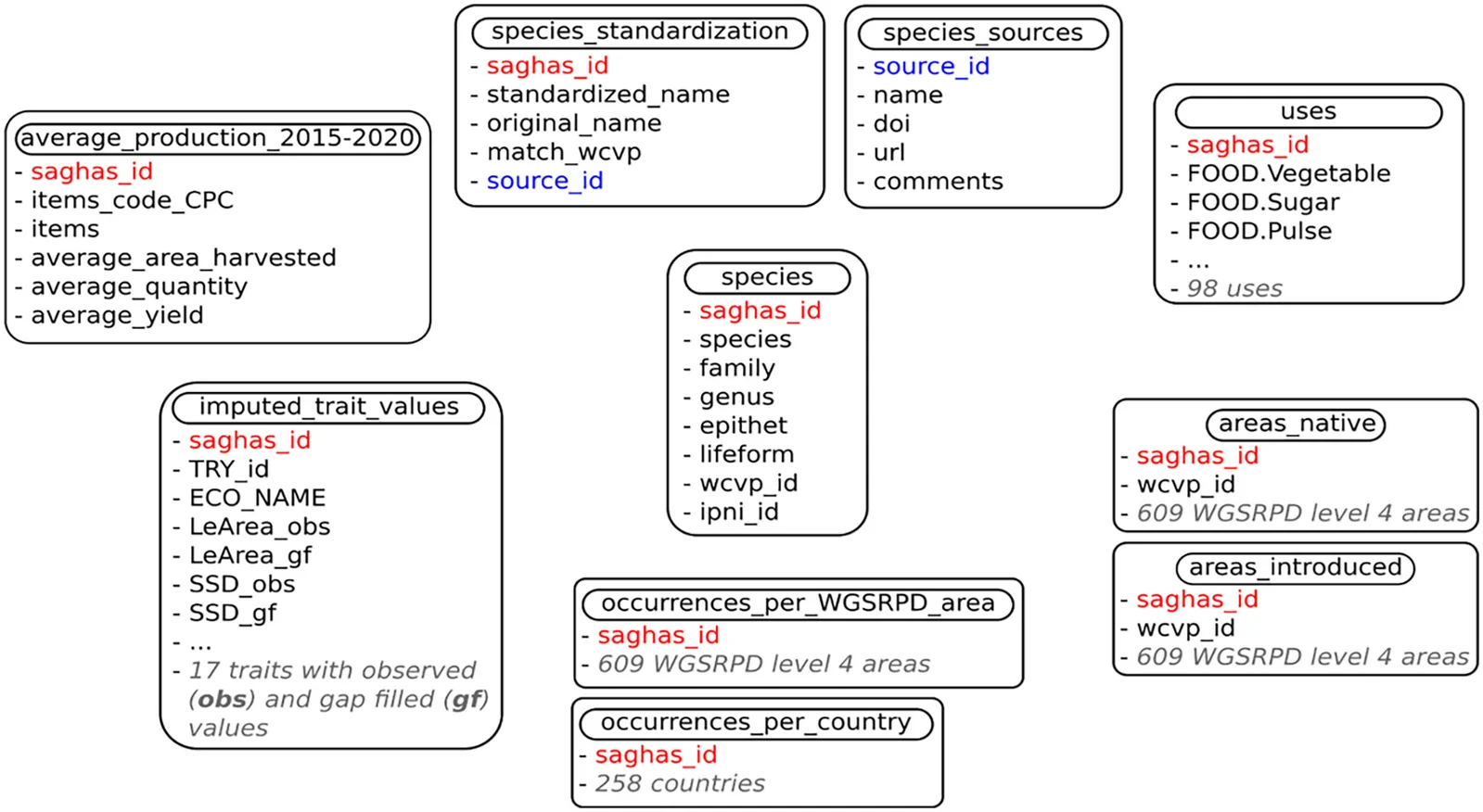
Dataset structure. Each table of the dataset is represented by a square, in which the column names are listed. In the center, “species” provides the list of species. Starting at the top and moving clockwise, the tables are: Standardization table of the species names, information on sources used to build the species list, human uses of plant species, native and introduced areas, number of occurrences per species and per area or country, functional trait values and production data. All tables are linked through the unique identifier “saghas_id”, in red, which refers to a species. The “species_standaridization” table is linked to “species_sources” via the source identifier “source_id”, in blue.

### 3.1 List of plants cultivated for food

We compiled a list of 2,449 plant species from 17 datasets (see Table 2 in this paper and table “species_sources” for detailed information), selecting only species defined as cultivated or domesticated for human consumption. In our dataset, three tables are related to the species list and the species sources.

The table named “species_sources” lists all of the 17 datasets used to compile the complete list, giving their names, DOI and/or URL to data, the related scientific publication when appropriate and comments indicating how to access the data. It also links each source to an identifier named “source_id”.

The table named “species_standardization” contains all the species names, original and standardized. In each row of this table, species “original name” is indicated as stated in the source (“source_id”) and linked to its “standardized name”. We standardized species names using the World Checklist of Vascular Plants (WCVP) database. Each standardized name is linked to a “saghas_id”, a unique identifier linking all the tables in the SAGHAS dataset.

The table “species” lists all standardized species names with taxonomic information on its family, genus and epithet. We also extracted life form information from the WCVP, which provides only a “broad and general” classification. The main categories are: tree, shrub, subshrub, perennial, geophyte, hydrophyte, and annual, with additional descriptors such as climbing, epiphytic, lithophytic, and parasitic.

Each species is associated with three identifiers: the “saghas_id”, the “wcvp_id” from the WCVP databases and resulting from the standardization process and an “ipni_id” from the International Plant Name Index (IPNI) also obtained during the standardization. WCVP IDs are linked to unique species names in the WCVP database, and the same applies for the IPNI IDs in the IPNI database. Both identifiers are useful to make connections with other datasets, as they refer to widely used taxonomic standards.

### 3.2 Compiled information on crops

The standardization process of the species list facilitated the compilation of various data on crop species. Therefore, we integrated information on human uses, native and introduced areas, number of occurrences per species and per area, functional trait values and production data to the SAGHAS dataset. Hereafter, we described the structure and the origin of those data.

#### 3.2.1 Human uses

This table provides information on human uses of the crop species listed in SAGHAS. There are 100 different uses in total, classified into 15 categories (Table 1). The first column of the table “uses” contains the species identifier (“saghas_id”) and the remaining 100 columns contain boolean values for each use, 1 indicates that a given species has been reported for a given use, while 0 indicates that it has not. NA values are assigned to species without any reported use and to uses with no associated species. Column names follow the format “<CATEGORY>.<use>”; for example “FOOD.Cereal_PseudoCereal” refers to the following use: “cereal_pseudocereal” of the “FOOD” category.

**Table 1:** List of human uses of crops per category sourced primarily from the Germplasm Resources Information Network (GRIN) database.

Category	Uses
FOOD	vegetable ; sugar ; starch ; pulse ; oil ; nut ; flavoring ; fruit ; cereal_pseudocereal ; beverage ; seeds
SOCIAL	hallucinogen ; masticatory ; narcotic ; smoking_material ; religious/secular ; psychoactive ; stimulant
MATERIAL	wood ; fiber ; lipids ; cane ; essential_oils ; tannin/dyestuff ; gum/resin ; wax ; latex/rubber ; carved_material ; beads ; chemicals ; alcohol ; cork/cork_substitute ; curiosity_item ; wrapping ; abrasive
FUEL	charcoal ; fuelwood ; petroleum_substitute/alcohol ; biomass_for_energy_generation ; miscellaneous_fuel ; biomass_for_energy_production
ENVIRON	ornamental ; agroforestry ; erosion_control ; soil_improver ; shade/shelter ; boundary/barrier/support ; revegetator ; lawn/turf ; pollution_control ; wildlife_habitat ; hemiparasite_host ; parasite_host_control ; firebreak ; host_for_edible_fungus
FORAGE	forage ; fodder
ADDITIVE	coloring ; tenderizer ; adulterant ; sweetener ; emulsifier ; stabilizer ; flavor_fixative ; thickening_agent ; gelling_agent ; preservative ; clarifier ; dietary_fiber ; fermentation_retarder
WEED	seed_contaminant ; parasite
MEDICINE	folklore ; source_of_pharmaceutical_agent ; veterinary ; delivery_system
PESTICIDE	plant_pest_control ; house_pest_control ; plant_disease_control ; weed_control ; disease_vector_control ; livestock_pest_control ; to_native_invertebrates
POISON	fish ; mammals ; birds
BEE	honey_production
HOST	dye-containing_insects ; lac_insects ; silkworms ; wax_insects ; edible_insects/caterpillars/larvae ; wood ; fiber
GENETIC	research_in_plant_biology
ALTHOST	crop_pests ; crop_diseases ; test_organism_for_crop_diseases

We sourced human use information from 14 of the 17 sources used to compile the species list, with the GRIN database being the major contributor of human use data. Table 2 indicates which sources provide information on human use.

**Table 2:** Sources used to compile the SAGHAS crop list. The table details the number of raw taxon names provided per source, the number of species standardized and selected per source and the number of species found exclusively in each source. The table gives criteria used to select them. “DOI / URL” column ideally provides the DOI of the related dataset, but it may also include a URL to the related dataset or the DOI/URL to the paper. It also indicated if the source was used to get species use information.

ID	Names	DOI / URL	Raw taxon names	Selected and standardised species	Source-exclusive species	Species selection criteria	Use source
africanOrphanCrops	African Orphan Crops Consortium [2]	https://africanorphancrops.org/	101	100	19	All species from this source are cultivated for human purpose	no
Clement1999	1492 and the loss of amazonian crop genetic resources. I. The relation between domestication and human population decline [3]	doi.org/10.1007/BF02866498	138	108	2	Species with at least one of these usages: “fruit”, “vegetable”, “root”, “condiment”, “cereal”, “oil”, “seed”, “nut”, “fruit juice”, “starchy fruit” and “oily fruit”	yes
ecocrop	ECOCROP	https://gaez.fao.org/pages/ecocrop	2568	491	73	Species mainly used as food and beverages, and reported as being grown on a small or large scale, or previously widely cultivated	yes
khoury2023	The Plants That Feed the World: Baseline data and metrics to inform strategies for the conservation and use of plant genetic resources for food and agriculture [4]	doi.org/10.5281/zenodo.8327962	536	337	11	Species with at least one of these usages : “Cereal”, “Fruit”, “Herb and spice”, “Nut”, “Oil”, “Pulse”, “Root and tuber”, “sugar”, “Vegetable”, “Beverage”	yes
gari2001	Biodiversity and Indigenous Agroecology in Amazonia [5]	https://www.researchgate.net/publication/284034435_Biodiversity_and_Indigenous_Agroecology_in_Amazonia_The_Indigenous_Peoples_of_Pastaza	53	35	4	Species mentioned as being used for food purposes	no
grin	GRIN-Taxonomy database - GRIN [6]	https://npgsweb.ars-grin.gov/gringlobal/downloads/default	79.933	1.138	340	Species marked as cultivated in table “taxonomy-geography_map.txt” and that have at least one usage in FOOD category except “gum/mucilage” or have flavoring usage in ADDITIVE category	yes
hernandez1992	Cultivos marginados otra perspectiva de 1492 [7]	https://openknowledge.fao.org/server/api/core/bitstreams/e1361dad-32ea-4a7b-92b0-905f188c43b4/content	258	211	34	Species with at least one of these usages: “Cereales y otros granos”, “Especias y condimento”, “Frutas y nueces”, “Hortalizas”, “Leguminosas de grano”, “Oleaginosas”, “Raices y tuberculo”	yes
jarvis2024	The Periodic Table of Food Initiative (PTFI): An initial list of the world’s food diversity for compositional investigation [8]	https://doi.org/10.7910/DVN/M9TA1H	1195	300	25	In this source, all species are reported as consumed by humans. We selected all species reported as cultivated	yes
levis2017	Persistent effects of pre-Columbian plant domestication on Amazonian forest composition [9]	doi.org/10.1126/science.aal0157	85	76	5	Species with at least incipient level of domestication and one of this usages: “Fruit”, “Edible nut”, “Starchy fruit”, “oily fruit”, “Flavoring”, “nut”	yes
li2018	Future smart food [10]	http://www.fao.org/3/I9136EN/i9136en.pdf	286	177	25	All species names mentioned in table from part 6 to 13 of the report	yes
meldrum2018	Issues and Prospects for the Sustainable Use and Conservation of Cultivated Vegetable Diversity for More Nutrition-Sensitive Agriculture [11]	doi.org/10.3390/agriculture8070112	1097	1085	397	All species from this source are cultivated for human consumption	yes
meyer2012	Patterns and processes in crop domestication: an historical review and quantitative analysis of 203 global food crops [12]	doi.org/10.1111/j.1469-8137.2012.04253.x	212	188	0	Species described as “domesticated”, or “semi-domesticated “and as “originally” or “currently used for food”	yes
milla2020	Crop Origins and Phylo Food: A database and a phylogenetic tree to stimulate comparative analyses on the origins of food crops [13]	https://github.com/rubenmilla/Crop_Origins_Phylo/	867	762	112	Species mentioned as being used for food purposes	yes
muller2006	Antes and Hoy DÍa: Plant knowledge and categorization as adaptations to life in Panama in the Twenty-First Century [14]	doi.org/10.1663/0013-0001(2006)60[321:AAHDPK]2.0.CO;2	80	39	3	Species used for human consumption and found in one of the following place	
“yards”, “gardens”, “farms”, “swiddens”, “riverbanks”	no						
toensmeier2020	Perennial vegetables: A neglected resource for biodiversity, carbon sequestration, and nutrition [15]	doi.org/10.1371/journal.pone.0234611	610	591	137	All species are cultivated (with statuses ranging from experimental to global crops) and all are used for human food purposes	yes
ulian2020	Unlocking plant resources to support food security and promote sustainable agriculture [16]	doi.org/10.1002/ppp3.10145	165	82	9	Species described as “mainly cultivated” or “wild and cultivated”	yes
williams2002	Global research on underutilized crops [17]	https://cgspace.cgiar.org/server/api/core/bitstreams/6988f6c9-82cc-4460-b320-4629f279ea94/content	196	140	10	Species with at least one of these usages: “cereal and pseudocereal”, “fruits and nuts”, “Oil seeds, “root and tuber”, “Starch/sugar”, “vegetable and pulse”	yes

### 3.3 Native and introduced areas

For each species, we separated information on their native and introduced areas in two tables named “areas_native” and “areas_introduced”, respectively. The status information was sourced from the WCVP. According to the WCVP, native refers to “areas where the plants have been present since before the last Ice Age or arrived by natural colonisation”. Introduced refers to “areas where plants were introduced accidentally or deliberately by humans, which includes archaeophytes, neophytes, alien, casual, invasive and naturalised plants but not cultivated ones or those merely planted in wild areas but not self-reproducing”.

In the SAGHAS table, native and introduced areas correspond to the World Geographical Scheme for Ordering Plant Distributions (WGSRPD), a standard developed by the Biodiversity Information Standards (https://www.tdwg.org/standards/) and used by the WCVP to define these areas. The WGSRPD standard divides the world at four different levels. The first level is continental with nine areas. The second one is subcontinental with 52 areas. The third level closely follows the political boundaries of countries, even if not exactly, with 369 areas (large countries are subdivided according to their administrative units). The fourth is the basic recording unit that subdivides all countries into administrative units with 609 areas.

We used the fourth WGSRPD level, with one column per area and another one for the species identifier “saghas_id”. For a given species a value of 1 indicates that it is native (or introduced depending on the table) in the corresponding area.

#### 3.3.1 Number of occurrences per area

The tables, “occurrences_per_WGSRPD_area” and “occurrences_per_country” provide the number of occurrences recorded for each species of the SAGHAS list per area of the fourth WGSRPD level and per country, respectively. We used one column per area/country and another one for the species identifier “saghas_id”. A numeric value indicates the number of occurrences of a given species in a given area/country.

Species occurrence data used to compile these tables were sourced from Pironon et al, 2024 [1], a dataset compiling and harmonizing occurrences from seven databases (GBIF, BIEN, RAINBIO, speciesLink, BioTIME,and Genesys Crop Wild Relatives) for roughly 36,000 plant species used by humans.

#### 3.3.2 Trait values

Original functional trait values compiled in SAGHAS come from the TRY Plant Trait database (https://www.try-db.org/). This database is filled with missing values (0,3% of the complete dataset is filled with data). Of our list of 2,449 crop species, 2,275 species appear in TRY, and trait data fill only 2.3% of the table. Through a statistical process of imputation, Joswig et al. 2022 [18] filled gaps of the TRY database, publishing a table with observed and gap-filled trait values for 17 traits of 21,386 species from different ecoregions. Ecoregions are defined as environmentally homogeneous areas, the 867 were described by Olson et al, 2002 [19]. Traits listed are: leaf area, stem dry mass per stem fresh volume (also called specific density (SSD) or wood density), leaf area per leaf dry mass (specific leaf area, SLA), leaf carbon (C) content per leaf dry mass, leaf nitrogen (N) content per leaf dry mass, leaf phosphorus (P) content per leaf dry mass, plant height, seed dry mass, seed length, number of seeds per dispersal unit, leaf nitrogen (N) content per leaf area, leaf nitrogen/phosphorus (N/P) ratio, leaf nitrogen (N) isotope signature (delta 15N), leaf fresh mass, stem conduit density, dispersal unit length, and wood vessel element length.

Similarly to the other tables of the SAGHAS database, the first column of the “imputed_trait_values” table contains the species identifier “saghas_id”, the second column stores the TRY identifier and the third stores the name of ecoregions. The other columns correspond to the 17 traits. For each trait, there are two columns: One containing observed values (suffix “_obs”) when available from TRY and one for imputed values (suffix “_gf”) imputed by Joswig et al. [18].

#### 3.3.3 Production data

We integrated the national statistics provided by the Food and Agricultural Organization (FAO) of the United Nations on quantity (in tons), cultivated area (in hectares) and resulting yields (in kg/ha) of crops to the SAGHAS database. Because the FAO provides data on items and not species (see method sections), the “average_production_2015-2020” table keeps track of the correspondence between datasets as follows: we use the “saghas_id” to identify crop species; “items_code_CPC” and “items” respectively identifier and name of FAO items. The three remaining columns are used to store the average produced quantity, cultivated area and yield calculated from the annual data between 2015 and 2020.

## 4 Materials and Methods

### 4.1 List of plants cultivated for food

#### 4.1.1 Sources selection

The construction of the SAGHAS database is based on the species list compiled by Khoury et al. (2023) as part of an FAO study on plant genetic resources for food and agriculture. This list provided a solid starting point with a total of 355 plant species, including all those covered in FAOSTAT (Annex 1 of the International Treaty), CGIAR mandate major crops, as well as other crops regarded as important at an international level. We then complemented this base with additional data sources: three databases and 14 published species lists known from the consortium. Our objective was not to conduct a formal systematic literature review. Rather than assembling individual articles on individual crops, we therefore targeted compiled lists — a category for which the number of English-language sources is relatively limited. We provide detailed information on all the datasets and publications used to build the SAGHAS database (see Table 2 in this paper and table “species_sources” in the published dataset). By combining multiple, complementary sources, we expect that the SAGHAS dataset captures a maximum of the documented diversity of food crops.

### 4.2 Species selection and standardization

We selected only species considered as cultivated or domesticated for human consumption by applying selection criteria for each source. Table 2 lists all the sources and selection criteria used.

We selected a total of 6,680 names, for which we needed to standardize the scientific names. We chose the WCVP standard maintained by Kew garden for its quality and stability over time. We used the function *wcvp_match_names()* from the R package *rWCVP* [20] to find matches between extracted scientific names with the standardized names from the WCVP (version 16, extracted on 04/06/2026), prioritizing exact matches, then fuzzy matches, and finally fuzzy phonetic matches. Fuzzy matching identifies names that differ slightly (e.g., due to misspellings). Fuzzy phonetic matching does the same but based on the phonetic transcription of names, which helps capture more spelling variations. To maximize the efficiency of the fuzzy process, we applied thresholds on the edition distance between original names and their matches:

• for fuzzy search: if the first letter is the same but the editing distance is greater than two then the match is rejected.
• for fuzzy search: if the first letter is different and the edition distance is greater than one then the match is rejected.
• for phonetic fuzzy search: matches with an edit distance greater than three are rejected.

After the standardization, we removed duplicated names. Only 147 names could not be kept due to absence of valid matches with WCVP species names: 102 of them did not exceed genus level, and 13 species, 4 hybrid species as well as 28 infraspecies (subspecies, variety of form) could not be standardized. Finally, we excluded seven species identified as toxic for human consumption according to the Plants For A Future website (pfaf.com). Fig. 3 shows the workflow leading to the final set of 2,449 species.

**Fig. 3:**
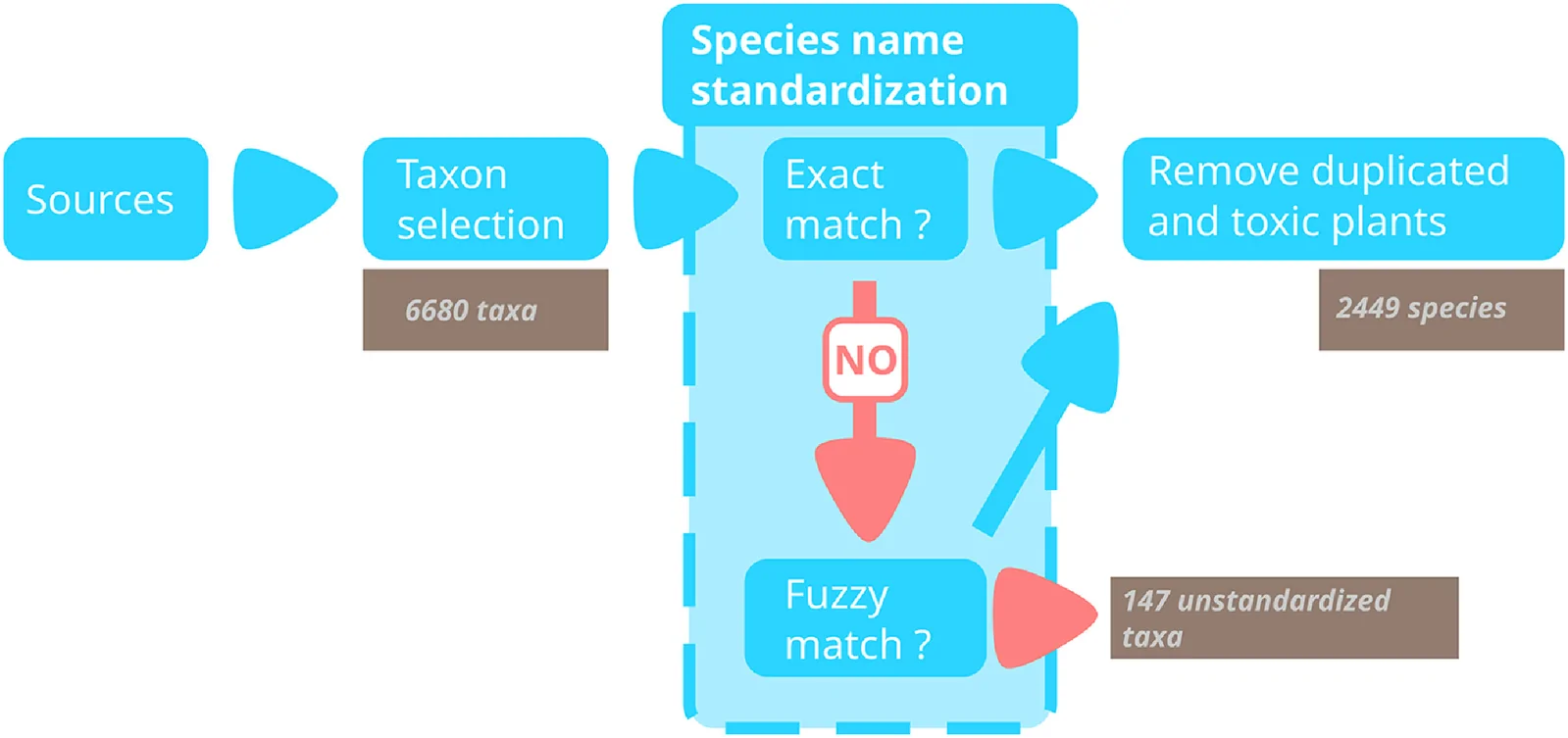
Workflow for the selection of crop species and standardization of the scientific names. For the “Taxon selection” step, specific criteria applied to each source are detailed in Table 2. The standardization of the species names was made using the WCVP (version extracted the 28/05/2025)

### 4.3 Compiled information on crops

#### 4.3.1 Human uses

The GRIN database was our primary reference for human uses of crops in our list. It classifies 103 uses into 15 categories adapted from the economic botany data collection standard. When available, we complemented information from GRIN with data provided by the sources used to compile the species list. To integrate information across sources, we introduced minor modifications to the GRIN human uses. We merged FOOD.cereal with FOOD.pseudocereal, FOOD.flavoring with ADDITIVE.flavoring and we removed FOOD.gum.mucilage since it is was directly linked to nutritional income. As a result of these changes, the number of uses has decreased to 100. When we found information on species’ use in multiple sources, we retained all the information to enrich the reported knowledge on the multiple human uses of crops. Uses reported for infraspecific taxa selected in sources (e.g., subspecies, varieties, forms) were attributed to the corresponding species level.

#### 4.3.2 Native and introduced areas

The WCVP database served as a reference for standardizing species names in our dataset. We also extracted native and introduced areas from this database, meaning that no additional correspondence was required to link areas with our species. As the WCVP defines areas at WGSRPD level 3, we converted them to level 4 using the correspondence information available in the level 4 shapefile provided by the TDWG GitHub repository (https://github.com/tdwg/wgsrpd).

#### 4.3.3 Number of occurrences per area

We used occurrence data from Pironon et al. [1]. While the WCVP standard was originally used to harmonize species names in that dataset, we standardized the names again using the same version of the WCVP applied to our species list to ensure compatibility, as the WCVP has been updated since the Pironon et al. [1] dataset’s publication. The version used was 16, extracted on 04/06/2026.

For each species in the SAGHAS database that was present in the Pironon et al. [1] dataset, we counted the number of occurrences per country and per WGSRPD level 4 area.

#### 4.3.4 Trait values

As the dataset of Joswig et al. [18] used TRY species names, to link this dataset with the SAGHAS species list, we first used the species name harmonization file provided by TRY (https://www.try-db.org/TryWeb/Data.php#100). For SAGHAS species names not found in this harmonization file, we used the Leipzig Catalogue of Plants (LCVP) standard via the R packages lcvpplant, as it is the main taxonomic standard used by TRY. We were able to attribute imputed trait values for 1,040 species of the SAGHAS dataset. From the 867 ecoregions, 271 are represented, with an average of 3.2 ecoregions per species.

#### 4.3.5 Production data

The national statistics compiled by the FAO annually (https://www.fao.org/faostat/en/#data/QCL) on food and agriculture are widely used in academic research on agriculture. However, all the data are provided for 300 items, an item corresponding to a group of plant or animal species or commodities. To link the crop list from SAGHAS through the “saghas_id” with the items from FAOstat, we developed a correspondence table to facilitate the interplay between these databases. For this purpose, we used the descriptive table of FAOstat items, specifically the french as it provides more detailed information on scientific names in the descriptive column than the english version. This table can be accessed at https://www.fao.org/faostat/fr/#data/QCL under “Définitions et standards” / “Produits”. We first extracted scientific names from raw text in the description columns with R using a regular expression and then we standardized names with WCVP standard. We were able to associate a total of 235 species of SAGHAS dataset with 141 items of the FAOstat list. In some cases, a single commodity corresponded to several species, while in other cases, a single species was matched to several commodities. For example, the item “Anise, badian, coriander, cumin, caraway, fennel and juniper berries, raw”, was linked to seven species by creating one row per species with FAOstat values. Similarly, *Allium cepa* was associated with two FAOstat items: “Onions and shallots, green” and “Onions and shallots, fry (excluding dehydrated)”, resulting in two rows in the table. For each combination of species/FAO item, we calculate the average values for the quantity produced, area harvested and yield from 2015 to 2020.

## Limitations

The selection of datasets/databases as sources was guided by the availability, coverage, and relevance rather than by a systematic protocol.

## Ethics Statement

The authors have read and followed the ethical requirements for publication in Data in Brief and confirm that the current work does not involve human subjects, animal experiments, or any data collected from social media platforms.

## CRediT Author Statement

**AB, ACT, CBS**, and **DR** contributed to the conceptualization of the study. **MD** were responsible for compiling the data. The original draft of the manuscript was written by **MD**, with all authors contributing to the review and editing.

## Data Availability

DataverseSAGHAS - Sowing AGrobiodiversity to HArvest Sustainability: a multi-source database of crop species cultivated for human food (Original data).

## References

[1] Pironon S. (2024). « The global distribution of plants used by humans datasets: list of utilised species, occurrence data and model outputs at 10 arc-minutes spatial resolution ». Zenodo.

[2] Hendre P.S. (2019). « African Orphan Crops Consortium (AOCC): status of developing genomic resources for African orphan crops ». Planta.

[3] Clement C.R. (1999). « 1492 and the loss of amazonian crop genetic resources. I. The relation between domestication and human population decline ». Econ. Bot..

[4] Khoury C.K., Sotelo S., Amariles D., Hawtin G. (2023). https://openknowledge.fao.org/handle/20.500.14283/cc6876en.

[5] Garí J. (2001). « Biodiversity and indigenous agroecology in Amazonia: the indigenous peoples of Pastaza ». Etnoecologica.

[6] « National Plant Germplasm System, United States Department of Agriculture-Agricultural Research Service - GRIN-Global ». 2025. [En ligne]. Disponible sur:. https://npgsweb.ars-grin.gov/gringlobal/search

[7] J. E. Hernández Bermejo and J. León, Cultivos Marginados otra Perspectiva de 1492. in FAO: Produccion y proteccion vegetal, no. 26. Consulté le: 11 mars 2025. [En ligne]. Disponible sur: https://openknowledge.fao.org/server/api/core/bitstreams/e1361dad-32ea-4a7b-92b0-905f188c43b4/content.

[8] Jarvis A. (2024). « The Periodic Table of Food Initiative (PTFI): an initial list of the world’s food diversity for compositional investigation ». Harvard Dataverse.

[9] Levis C. (2017). « Persistent effects of pre-Columbian plant domestication on Amazonian forest composition ». Science.

[10] Li X., Siddique K.H.M. (2018). Future Smart Food: Rediscovering Hidden Treasures of Neglected and Underutilized Species for Zero Hunger in Asia.

[11] Meldrum G., Padulosi S., Lochetti G., Robitaille R., Diulgheroff S. (2018). « Issues and prospects for the sustainable use and conservation of cultivated vegetable diversity for more nutrition-sensitive agriculture ». Agriculture.

[12] Meyer R.S., DuVal A.E., Jensen H.R. (2012). « Patterns and processes in crop domestication: an historical review and quantitative analysis of 203 global food crops ». New Phytol..

[13] Milla R. (2020). « Crop Origins and Phylo Food: a database and a phylogenetic tree to stimulate comparative analyses on the origins of food crops ». Glob. Ecol. Biogeogr..

[14] MÜller-Schwarze N.K. (2006). « Antes and Hoy DÍa: plant knowledge and categorization as adaptations to life in Panama in the Twenty-First Century ». Econ. Bot..

[15] Toensmeier E., Ferguson R., Mehra M. (2020). « Perennial vegetables: A neglected resource for biodiversity, carbon sequestration, and nutrition ». PLoS One.

[16] Ulian T. (2020). « Unlocking plant resources to support food security and promote sustainable agriculture ». Plants People Planet.

[17] Williams J.T., Haq N. (2002).

[18] Joswig J.S. (2022). « Climatic and soil factors explain the two-dimensional spectrum of global plant trait variation ». Nat. Ecol. Evol..

[19] Olson D.M. (2001). « Terrestrial ecoregions of the world: a new map of life on Earth ». BioScience.

[20] Brown M.J.M. (2023). « rWCVP: a companion R package for the World Checklist of Vascular Plants ». New Phytol..

